# The Experience of Accessing Primary Healthcare Centres in a Lebanese Community: Perspectives of Older People, Family Members and Service Providers

**DOI:** 10.1111/hex.70449

**Published:** 2025-09-27

**Authors:** Saydeh Dableh, Kate Frazer, Mathilde Azar, Thilo Kroll

**Affiliations:** ^1^ School of Nursing, Midwifery, and Health Systems University College Dublin Dublin Ireland; ^2^ Nursing Program University of Balamand Beirut Lebanon

**Keywords:** experiences, healthcare accessibility, older people, people‐centred care, primary healthcare, qualitative design

## Abstract

**Introduction:**

Older people's experiences with access to primary healthcare are overlooked in LMICs, leading to inequitable access and limited delivery of person‐centred care. In Lebanon, the economic crisis has increased older people's vulnerabilities and reliance on services provided through primary healthcare centres (PHCCs). This study explores (1) factors shaping decisions of using PHCCs; (2) experiences of older people accessing PHCCs from three perspectives: the older people themselves, family members and service providers; (3) family members' experiences with accessing PHCCs; and (4) service providers' experiences with providing care for older people within PHCCs in a Northern Lebanese district.

**Methods:**

This study adopts a qualitative descriptive design with an inductive content analysis approach. Data were collected through seven focus group discussions and 15 individual interviews (*n* = 57 older people, family members, and service providers).

**Results:**

Factors shaping decisions of using PHCCs' services include socio‐economic status, knowledge of services, influences of family members, perceived service quality and proximity, age‐related changes, and providers' attitudes and behaviours. Older people reported varied access experiences shaped by factors at individual, organisational, communal, governmental and global levels. Positive experiences included enhanced autonomy, gratitude for receiving needed services, perceived care quality and socialising opportunities. However, negative experiences included humiliation and discomfort, anxiety, dependency, perceived status regression, perceived poor quality and a sense of being a burden. While some family members reported relief from getting affordable care, others reported discomfort, perceived status regression, blame for neglect and challenges with coordinating care across multiple providers. Service providers' experiences included pride in supporting older people, but resentment due to unfair remuneration.

**Conclusion:**

Findings reveal aspects of care that older people and family members appreciate and others that contribute to negative experiences. Experiences of older people, family members and service providers are interconnected. Quality improvement requires comprehensive approaches addressing their needs. Findings inform practitioners and policymakers to design multidimensional and people‐centred approaches to maximise healthcare access.

**Patient and Pubic Contribution:**

No PPI engagement methods were applied in this study or analysis. However, the findings informed discussions with older people and facilitated partnerships to co‐design a follow‐up study focused on developing solutions.

## 1 Introduction

With the increased focus on person‐centred approaches, understanding experiences with healthcare is becoming vital for generating innovative solutions and bridging healthcare gaps [1]. The human experience in healthcare ‘integrates the sum of all interactions, every encounter among patients, families, care partners and the healthcare workforce’ [2, p. 10] at different touchpoints across the continuum of care. In addition to organisations and patients' characteristics [1], environmental factors are highlighted for their influence on experiences with healthcare [2]. They shape patients' and families' needs, socio‐economic status, values and help‐seeking behaviours. They also drive organisational culture, priorities, and strategies affecting the delivery of services and the dynamics among the workforce, which impact the encounters between service users and providers.

Gaining a clear understanding of how older people perceive and experience healthcare services provided by primary healthcare centres (PHCCs) is crucial, especially as the ageing population continues to grow and increasingly relies on primary healthcare (PHC) services. Enhancing patients' experiences is correlated with improved quality of care, promoted patient engagement and adherence to treatment regimens, enhanced organisational performance, increased providers' satisfaction, and better health outcomes [3, 4]. Low‐ and middle‐income countries (LMICs), comprising the majority of the global older population, require further attention [5, 6]. Access to PHC is key to ensuring UHC and improving health outcomes [7]; however, available literature suggests that older people's experiences with access to PHC are poorly examined in LMICs and vary considering the local context, healthcare system characteristics, and older people's attributes [8, 9, 10]. Moreover, the characteristics, availability, attitudes and behaviours of PHC providers are identified as essential factors shaping older people's experiences with access to PHC [8].

### 1.1 The Lebanese Context

Lebanon is a middle‐income country with older people accounting for 11% of its total population [11]. Since 2019, the country has been grappling with a compounded crisis, steering the healthcare system to a total collapse [12, 13]. The Syrian crisis, the Covid‐19 pandemic, the Beirut blast and the unprecedented economic crisis particularly strained the healthcare system, with a notable shortage of financial resources and equipment [14] and a massive immigration of physicians and nurses [15]. Main characteristics of the healthcare system apply to the PHC sector in the country; it is highly privatised, fragmented and therapeutic‐care oriented because of the nominal fund dedicated to preventive care (only 5% of the Lebanese Ministry of Public Health [MOPH] budget) [16]. Due to a shortage of geriatric wards and specialists [11], general practitioners and nurses mainly deliver care to older people. This is not expected to improve due to providers' immigration [15].

As multiple crises accumulate and challenge the living in Lebanon, older people may be particularly vulnerable to financial, health and social stressors. More than 50% of them do not benefit from health coverage and social pension plans. They rely on their families to get needed services, which may result in delayed care [17] and interference of family members in healthcare‐seeking decisions [11]. Evidence from Lebanon identified 75% of older people living with at least one disease, and 25% reported having a poor health status [18].

Lebanese citizens have always preferred to seek primary care from private providers [19, 20], rather than from the PHC network (300 PHCCs in 2023), established by the MOPH to deliver a package of affordable PHC services across the country [21, 22]. A shift in healthcare‐seeking behaviours towards an increased use of PHCCs, by 97% in 2023 compared to 2019, was noted after the economic recession [22], while experiences of older people with access to those centres are still unexplored.

Therefore, this study aims to (1) identify factors influencing older people's decisions to use services delivered at PHCCs; (2) explore experiences of older people accessing PHCCs from three perspectives: the older people themselves, family members and service providers; (3) explore family members' experiences of accessing PHCCs; and (4) explore service providers' experience of delivering PHC to older people within PHCCs. While this study focuses on older people's experiences with access to PHCCs, it acknowledges the inherent influence of family members and service providers' experiences.

## 2 Methods

### 2.1 Study Setting

We conducted this study in Koura, a district in the North Lebanon governorate, selected for being a peripheral area lacking specialised care for older people. Koura district is comprised of mostly rural villages with urban centres and 84,600 people from varying cultural backgrounds, reflecting the mixed Lebanese community [23]. While older people account for 10.9% of the population in Koura, 78.5% of households report an average or poorer economic status, affecting their access to PHC, particularly in rural areas of the district [23]. Healthcare in Koura shares similar challenges and characteristics with the broader Lebanese healthcare system. Residents are served by a mix of public, private and non‐governmental institutions, including five PHCCs operating according to the Lebanese national PHC standards, three hospitals, and several dispensaries and private clinics. For this study, we selected two PHCCs representing different funding sources (internal vs. external) and staff mix composition, along with varying size and cultural background of beneficiaries.

### 2.2 Study Design

This descriptive qualitative study, conducted from a pragmatic lens, involved older people as users and non‐users of PHCCs' services, family members and service providers, to capture different perspectives and experiences [24]. While users are expected to provide their actual experiences, non‐users may highlight constraining factors. The insights of family members are essential as they significantly support older people in their healthcare decision‐making [17]. Service providers highlight system‐based factors that shape older people's decisions and experiences. This multi‐perspective approach enriches the study and allows for triangulation of data sources, resulting in a comprehensive understanding of older people's access to PHCCs.

Qualitative descriptive designs are widely used in healthcare and service research as they provide a comprehensive summary of events, which is particularly useful for practitioners and policymakers [25, 26]. They generate rich and straightforward descriptions while staying close to the data and participants' language [27]. This research design aligns with the pragmatic focus of this study to generate actionable knowledge that informs future practice [24]. It is particularly suitable since knowledge of the topic in Lebanon is limited. Moreover, there is a pressing need to understand and address access issues amid combined crises exacerbating older people's vulnerabilities [11]. To ensure reporting rigour, this manuscript follows the Standards for Reporting Qualitative Research (SRQR) [28].

### 2.3 Ethical Considerations

This study was approved by the institutional research board of the University of Balamand in Lebanon (IRB‐REC/O/023‐23l0923, July 2023) and the health research ethics board of the University College Dublin (LS‐CO‐23‐185‐Dableh‐Kroll, 23 November 2023). All participants provided a signed informed consent. They were informed about data confidentiality and security measures.

### 2.4 Participants and Recruitment

Older people who used PHCCs' services (designated as users or UP), family members of users (designated as FMU) and service providers (designated as SP) were recruited through the two selected PHCCs in Koura. Older people who did not use PHCCs' services (designated as non‐users or NUP) and family members of non‐users (designated as FMNU) were recruited through municipalities where the two PHCCs were located. Participants eligible to take part in this study were (1) Lebanese adults aged 60 years and older, using different PHCCs' services during the past year or not using PHCCs' services, and able to provide consent for themselves; (2) close relatives who are the main caregivers for users and non‐users; and (3) service providers including administrators and health professionals with at least 1 year of professional experience with the selected PHCCs and serving older people. Older people living in residential homes were excluded as they receive PHC through those institutions. Eligible individuals were informed about the study through focal people at selected PHCCs and municipalities. Those who were interested contacted the principal investigator (PI), who recruited participants on purpose to ensure maximum variation considering the use of PHCCs services, socio‐economic status, age and gender criteria. Two criteria were used to determine the sample size: (1) authors' judgement on the achievement of adequate data to answer the research questions and develop themes [29], alongside (2) code saturation (no new codes) and meaning saturation (no iterative descriptions to codes listed in the codebook) [30]. Table 1 outlines the application of these two criteria across participant categories to determine the sample size.

**Table 1:** Determining the sample size.

	Participant categories			
Criteria for decision	Older people users	Older people non‐users	Family members	Service providers
Authors' judgement on data adequacy	✔	✔	✔	✔
Code and meaning saturation	✔	✔	✔	✔
	Achieved at 2 FGDs and 5 interviews (the additional 3 interviews didn't yield new insights)	Achieved after 1 FGD and 4 interviews (the additional 2 interviews didn't yield new insights)	Achieved at the 1 FGD and 1 interview (the second FGD didn't yield new insights)	Achieved at the first FGD (the second didn't yield new insights)

Abbreviation: FGD = focus group discussion.

### 2.5 Data Collection

The PI (S.D.) collected data between November 2023 and January 2024, through focus group discussions (FGDs), supplemented by individual interviews with older people who presented motor and sensory limitations impeding their participation in FGDs. FGDs took around 90 min and were conducted either at PHCCs or at an accessible venue for all participants, while the 20‐ to 45‐min interviews took place at participants' homes. All participants were asked about their views regarding (1) older people's experiences with access to PHCCs and (2) factors and perceptions shaping older people's decisions to use services delivered at PHCCs. Family members were also asked to describe their experiences of accessing PHCCs. Service providers were additionally asked to reflect on their experiences with providing care for older people within PHCCs. The data included socio‐demographic characteristics (Table 4) and were collected in Arabic and audio‐recorded according to the provided consent.

### 2.6 Data Analysis

Simultaneously with data collection, the PI manually transcribed the audio recordings verbatim, reviewed the manuscripts and noted impressions, which facilitated a deeper immersion in the data. To involve non‐Arabic speaking co‐authors in data analysis, 3 transcripts out of 22 were translated into English, consisting of one FGD manuscript per participant category, to provide broad data. At the early coding stages, S.D., T.K. and K.F. independently coded the translated transcripts and then met to discuss and ensure that all relevant aspects were captured. Given the high inter‐coder agreement, the authors decided to continue the analysis of the remaining data in Arabic to conserve the meaning carried by participants' language, which enhances data trustworthiness [31]. The PI also translated an extensive list of quotes into English (Supporting Material 1) to facilitate audit and rigour in the analysis process. Table 2 details the data translation and back‐translation processes used through different research stages.

**Table 2:** Data translation.

Research phase	Translation/research activity	Objective	People involved
Before data collection	Translating the study instruments into Arabic.	Collecting data in the language of participants.	S.D., a bilingual author
	Checking the quality of translation, discussing with S.D. and elaborating the final Arabic versions.	Enhancing the quality of forward translation.	M.A., a bilingual author
After data collection	Analysing most data in Arabic by applying English codes to Arabic transcripts	Conserving the meaning carried by the participants' language.	S.D.
	Translating into English, 3 of 22 transcripts, representing 1 FGD manuscript for each participant's category.	Facilitating the peer debriefing process.	S.D.
	Translating an extracted list of metaphors, jargon, idioms and quotes.	Enhancing the quality of forward translation and engaging co‐authors in discussions.	S.D. and a professional translator
	Back‐translating four random English pages into Arabic; Reviewing translation processes to ensure that capture of both language nuances and health research vocabulary was accurate.	Enhancing translation quality.	A Lebanese, doctoral‐level, senior administrator in UCD, affiliated to the School of Nursing, Midwifery, and Health Systems, independent from the authors, and immersed in both cultures.
	Comparing the back‐translated version against the original Arabic version and then meeting to discuss and analyse translation discrepancies and reach a consensus about major technical terms.	Enhancing translation quality and finalising English transcripts.	S.D. and the Lebanese staff member affiliated with the School of Nursing, Midwifery, and Health Systems at UCD
	Translating the Arabic quotes into English.	Facilitating dissemination.	S.D., expert translator, a Lebanese‐American friend

Abbreviations: FGD = focus group discussion, UCD = University College Dublin.

An inductive content analysis approach was employed as it best aligns with qualitative descriptive designs to provide rich data summaries [25, 26, 27]. Analysis steps as described by Erlingsson and Brysiewicz [32] were followed. The PI (S.D.) condensed transcripts into meaning units consisting of words or sentences conveying essential information regarding the research questions. Meaning units were coded in each transcript and then charted into a spreadsheet, with columns showing codes and rows showing acronyms corresponding to the different participant categories and data collection methods (e.g., SP‐FGD2). Iterative coding was applied to ensure that newly identified codes are not missed in transcripts coded earlier. Codes were sorted into eight categories (Table 3) related to research aims. Initial codes and descriptors were iteratively updated (Supporting Material 2) and shared with co‐authors for discussion and further refinement. Through collaborative discussions among co‐authors, themes were then developed expressing the underlying meaning of codes [32]. To facilitate data triangulation, a different spreadsheet was used to map codes under each category (columns), and across different participants and data collection methods (rows). This multistage approach to analysis allowed us to summarise, then compare and contrast data to reflect different viewpoints across participant categories, and present rich descriptions. After removing personal data identifiers, the second bilingual co‐author (M.A.) verified the coding of meaning units, the generation of categories, and data mapping. Accordingly, S.D. and M.A. discussed and made iterative changes. The extensive list of translated quotations (Supporting Material 1) was used to enhance the confirmability of the PI's interpretation. Table 3 provides an example of the coding process leading to the generation of themes.

**Table 3:** Example of the coding process.

Meaning units	Example of codes	Categories	Themes
*I feel ashamed to add to his burdens (UP)*	Feeling of being a burden	Personal factor	Perceptions and factors shaping older people and family members' decisions to seek care from PHCCs
*Older people are left to their unknown fate. The government should take care of us (UP)*	Perceived lack of governmental support	Contextual factor	
*We are grateful for getting the medications that we need (UP)*	Gratefulness for the received service	Positive older people's experience	Accessing PHCCs: A gateway to restored dignity and well‐being
*It could be a disaster without the services provided by the centre at low cost (FMU)*	Relief to get affordable care	Positive family members' experience	
*We are proud of being able to help older people (SP)*	Pride to support older people	Positive service provider experiences	
*Service quality is below zero (UP)*	Perceived low quality	Negative older people's experience	The burden of free care delivered at PHCCs
*I am leaving my old‐aged mom alone (FMU)*	Discomfort	Negative family members' experience	
*It's difficult to make a diagnosis (SP)*	Difficulty to assess	Negative service provider's experience	

Abbreviations: FMU = family member of user, SP = service provider, UP = user participant.

### 2.7 Rigour and Trustworthiness

Various strategies were implemented to ensure research rigour [33], including prolonged engagement with data, data collection by the same researcher across stakeholders, peer debriefing, and detailed contextual descriptions. The credibility of findings was enhanced by triangulating data from different sources and data collection methods, analysing data in the native language of the participants to preserve the context‐specific meaning and cultural nuances of their perspectives, independent coding of transcripts (3 out of 22), verification of the coding process by a bilingual co‐author, documenting the data translation process [31], and reporting an extensive list of quotes to substantiate the findings. Additional measures included maintaining an audit trail and keeping a reflexive journal. The study was conducted by experienced researchers specialising in qualitative designs (T.K., K.F. and M.A.) and PHC in Lebanon (S.D.). The PI's expertise in PHC and geriatric care shaped the research focus and methodology. To ensure unbiased responses, participants were recruited through gatekeepers with whom the investigator had no prior relationship.

## 3 Results

### 3.1 Participants' Description

**Figure 1:**
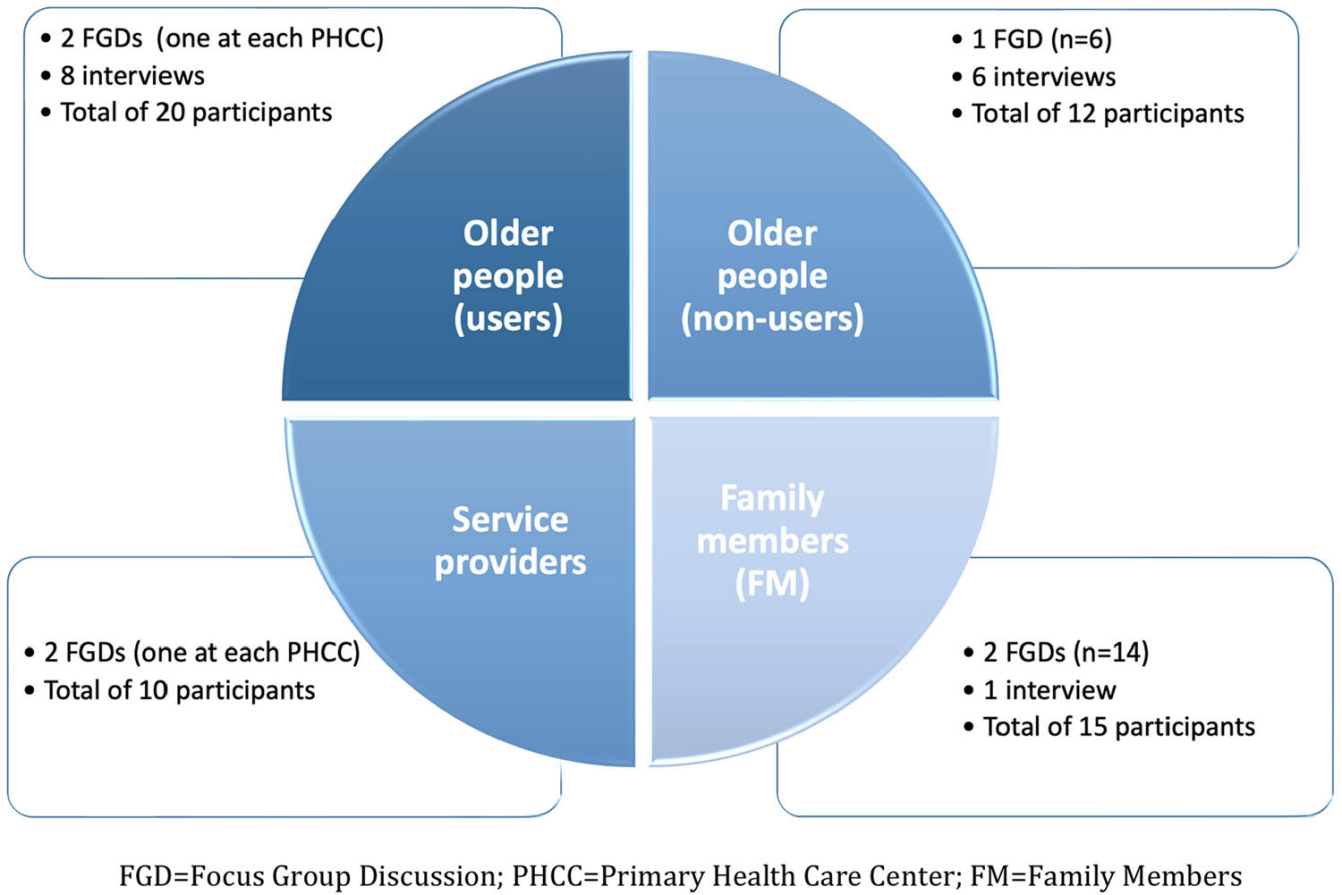
Data collection.

Seven FGDs and fifteen individual interviews were completed, and the details are demonstrated in Figure 1.

Table 4 describes the varied participants' socio‐demographic characteristics. It is worth noting that 91% of participating older people present at least one chronic disease, and 41% live with at least one functional limitation. However, 47% of them lack health coverage, and 97% rate their socio‐economic status as low to medium.

**Table 4:** Participants' characteristics.

	Older people (*n* = 32)	Family members (*n* = 15)	Service providers (*n* = 10)
Age	60–70: 31% (*n* = 10) 71–79: 53% (*n* = 17) ≥ 80: 16% (*n* = 5)	30–40: 20% (*n* = 3) 41–50: 27% (*n* = 4) 51–65: 53% (*n* = 8)	
Gender	Female: 53% (*n* = 17) Male: 47% (*n* = 15)	Female: 67% (*n* = 10) Male: 33% (*n* = 5)	Female: 70% (*n* = 7) Male: 30% (*n* = 3)
Occupation	Currently working: 6% (*n* = 2) Not working: 94% (*n* = 30)	None: 54% (*n* = 8) Private sector: 33% (*n* = 5) Public sector: 13% (*n* = 2)	
Education	None: 3% (*n* = 1) Primary–secondary: 75% (*n* = 24) University: 22% (*n* = 7)	None: 0% (*n* = 0) Primary–secondary: 47% (*n* = 7) University: 53% (*n* = 8)	None: 0% (*n* = 0) Primary–secondary: 20% (*n* = 2) University: 80% (*n* = 8)
Socio‐economic status (self‐rated)	Low: 53% (*n* = 17) Medium: 44% (*n* = 14) High: 3% (*n* = 1)	Low: 47% (*n* = 7) Medium: 53% (*n* = 8) High: 0% (*n* = 0)	
Health insurance^a^	Public: 47% (*n* = 15) Private: 9% (*n* = 3) None: 47% (*n* = 15)	Public: 53% (*n* = 8) Private: 20% (*n* = 3) None: 27% (*n* = 4)	
Chronic disease	None: 9% (*n* = 3) 1–2: 56% (*n* = 18) ≥ 3: 35% (*n* = 11)		
Functional limitation (self‐reported)^b^	None: 66% (*n* = 21) Cognitive limitation: 0% Sensory limitation: 19% (*n* = 4) Motor limitation: 22% (*n* = 7)		
Role at the PHCC			Physician: 20% (*n* = 2) Nurse/nurse assistant: 40% (*n* = 4) Non‐medical staff: 10% (*n* = 1) Administrative: 30% (*n* = 3)
Years of experience at PHCCs			Up to 10 years: 50% (*n* = 5) More than 10 years: 50% (*n* = 5)

^a^ One participant can have both public and private insurance.

^b^ One participant can have multiple types of limitations.

The findings are presented according to the three generated themes. Theme 1 fulfils the first research aim by describing perceptions and factors shaping older people's decision to seek care from PHCCs. While the second theme groups positive experiences expressed by older people, family members and service providers, the third reflects their negative experiences. Themes, categories, codes and supportive quotes are listed in Supporting Material 1.

### 3.2 Theme 1. Perceptions and Factors Shaping Older People's Decision to Seek Care From PHCCs

Participants mentioned several personal and contextual factors shaping people's decisions regarding the use of services delivered through PHCCs.

#### 3.2.1 Personal Factors

##### 3.2.1.1 Misconceptions About the PHC Network

Older people and family members highlighted a knowledge gap negatively affecting their decision to access PHCCs. While some participants acknowledged their unfamiliarity with the PHC concept, others mistakenly included tertiary and secondary care services, such as hospitalisations and surgeries, within primary care. Moreover, many participants, even service providers, used the terms PHCCs and dispensaries interchangeably, with dispensaries being the familiar term.

I never heard about primary health care.

UP, female, 60–70 age group

Dispensaries are more common, I am not sure if primary health care centres exist in Lebanon.

FMNU, female, 51–65 age group

##### 3.2.1.2 Perceived Changes in Circumstances

Some older people highlighted age‐related changes, such as illness, partner's death and severe financial shortages, that support their decision to use nearby PHCCs' services, requiring less mobility and transportation. The economic crisis, particularly, shifted healthcare‐seeking behaviours towards more use of PHCCs' services but less use of preventive care.

I use the primary care centre because my husband passed away and his pension is stuck in the bank. I cannot even afford a loaf of bread, I am ruined!

UP, female, ≥ 80 age group

Many participants across categories reported that older people are more inclined to use PHCC services after the economic crisis; normalisation of use is replacing stigmatisation of users.

##### 3.2.1.3 Perceived Difference Between PHCCs and Private Clinics

While several users preferred PHCCs over private clinics for being more affordable, easily accessible, with more friendly and caring providers, others reported that private clinics deliver better‐quality care, especially in terms of medical examination with reduced waiting time and overcrowding. Participants stated that examinations at PHCCs are rushed, and providers dedicate more time at private clinics to care, discuss and explain.

At primary care centres, they do what is strictly necessary; if you present high blood pressure, they measure it and prescribe medications. Questions about when it happened and how it happened do not exist!

FMU, female, 51–65 age group

The perceived difference in the quality of care delivered in the PHCCs and private clinics determines participants' choices of the care setting.

##### 3.2.1.4 Shared Decision‐Making

Several older people reported a delegation of the care setting choice to their family members who pay for healthcare services. Many older people who do not use PHCCs' services reported the influence of their children, who recommend private providers for enhanced care and social image.

I called my son to discuss with him my neighbour's recommendation for seeking services provided by the nearby centre, but he totally refused.

NUP, Female, 71–70 age group

#### 3.2.2 Contextual Factors

##### 3.2.2.1 Perceived Consequences of the Economic Crisis

All participants flagged that the economic crisis is limiting older people's financial abilities to afford care. This is due to the collapse of savings at banks, currency devaluation and dysfunctional public insurance schemes. Several participants stated that they started accessing PHCCs after the economic crisis, seeking chronic medications that either became unavailable at pharmacies or were very expensive. Contextual factors have implications on the personal level; many older people who used to be financially independent rely fully on their children and are obliged to seek services from PHCCs.

Mr. the army commander and I have the same case, we used to have 100% health coverage and many other benefits; we should not have been obliged to come to the primary care centre, they are humiliating us after all these years.

UP, male, 60–70 age group

##### 3.2.2.2 Perceived Lack of Governmental Support

Many older people agreed that they are obliged to use PHCCs' services, given the lack of governmental policies preserving their basic rights. People who used to work within the private sector stated that they paid taxes and lost their health insurance at retirement age, when they mostly needed healthcare. Older people contrast their status with peers living abroad and blame the government for lacking pension plans, health coverage, transportation assistance and prescheduled regular check‐up appointments, which impede their access to healthcare, especially to private settings.

In Lebanon, older people are left to their unknown fate. The government should take care of us. We deserve at our old age to get decent services.

NUP, male, 60–70 age group

### 3.3 Theme 2. Accessing PHCCs: A Gateway to Restored Dignity and Well‐Being

Participants across categories reported mixed experiences regarding either using or delivering services through PHCCs. This theme reflects positive experiences with different perspectives.

#### 3.3.1 Positive Experiences of Older People

Several older people using PHCCs shared positive perceptions and considered that using such services constitutes a choice because of multiple factors, as described in Table 5. Service providers and family members also reflected on older people's positive perceptions. Users of the externally funded PHCC reported more positive experiences, particularly regarding service availability and quality, along with providers' attitudes, compared to users of the PHCC receiving local funds.

**Table 5:** Positive experiences of older people accessing PHCCs.

Perception/feeling	Contributing factor	Supportive quotes
Enhanced autonomy	Proximity; Independent access; Ease of making appointments	‘*I prefer going to the health centre, it is nearby and I can go alone’* (UP, female, 71–79 age group) ‘*People living nearby walk to the centre; however, it is not that easy for people coming from surrounding villages’* (SP, up to 10 years of experience) ‘*They can book appointments by just calling’* (FMU, female, 30–40 age group)
Perceived good service quality	Availability of care that responds to their perceived need;	‘*The quality of services is as good as those delivered through private clinics’* (UP, female, 60–70 age group)
	Care provider's competence;	‘*They trust our services because attending physicians are well‐known and have their private clinics’* (SP, up to 10 years of experience)
	Patient preparation is part of the physical examination.	‘*As in private clinics, a nurse prepares the older patients and accompanies them to the physician who dedicates appropriate time’* (FMU, female, 41–50 age group)
Gratefulness for the received support and services	Availability of chronic medications and home care;	‘*Older people verbalize that they are grateful for getting needed services’* (SP, up to 10 years of experience)
	Supportive paramedical equipment; Periodic tests; Service cost.	‘*A physician and a nurse come to my home and bring medications for free; otherwise, I would not be able to get care’* (UP, female, 71–79 age group)
Being respected, heard and cared for	Welcoming and caring staff; Effective communication;	‘*People at primary care centres are very good and friendly. They respect older people specifically*’ (UP, female, 71–79 age group)
	Equal treatment of people; Organised appointments;	‘*They like to come and share their stories and personal issues, we listen to them and try to provide support’* (SP, up to 10 years of experience)
	Prioritising older people, especially those presenting specific cases and limited mobility.	‘*Once they get beneficiaries using a wheelchair, they give them a pass; they do not wait a minute’* (FMU, female, 41–50 age group).
Opportunity to socialise	Simple processes;	‘*I go there frequently, I got familiar with the staff and the process’* (UP, female, 71–79 age group)
	Familiarity with staff members.	‘*They share with us personal stuff that they do not even share with their children’* (SP, more than 10 years of experience)

Abbreviations: FMU = family member of users, SP = service providers, UP = user participants.

#### 3.3.2 Positive Experiences of Family Members

While reflecting on their experiences, some family members reported a sense of relief for getting affordable care for their parents, reducing their financial hardship, especially after the economic crisis.

It could be a disaster without services provided by the centre at low cost.

FMU, male, 30–40 age group

They also enjoyed positive relationships and highlighted the importance of providers' respectful attitudes contributing to their well‐being and recurrent use of services.

It really makes a difference when staff treat you respectfully.

FMU, female, 30–40 age group

#### 3.3.3 Positive Experiences of Service Providers

Many service providers reported pride in supporting older people during crisis times. Despite feeling drained, they stated that the gratitude manifested by older people and families was rewarding and increased their determination to deploy more efforts.

We are really proud, despite all challenges and short resources, we managed and we helped older people who had no one, nothing! Their grateful attitude was rewarding.

SP, up to 10 years of experience

### 3.4 Theme 3. The Burden of Free Care Delivered at PHCCs

This theme describes negative care experiences of older people, family members and service providers.

#### 3.4.1 Negative Experiences of Older People

Many older participants agreed that using PHCCs' services is an obligation in the aftermath of the economic crisis, in the absence of alternatives. This was supported by family members and service providers who also reflected on older people's experiences. Participants highlighted negative perceptions inherent to seeking free or affordable care at PHCCs and interpreted underlying factors (Table 6).

**Table 6:** Negative experiences of older people accessing PHCCs.

Perception/feeling	Contributing factor	Supportive quotes
Perceived low quality	Low cost; Superficial medical examination; Perceived physicians' low competence; Physicians' conduct and communication skills; Shared negative experiences.	‘*It seems that the service quality is not zero, it's below zero’* (UP, male, ≥ 80 age group) ‘*The physician is seeing around 40 patients in 1 h or 1 h and a half; he deals with you as a number’* (NUP, female, 71–79 age group) ‘*People think that the service quality is low because it is free’* (SP, more than 10 years of experience)
Feeling of being a burden	Financial dependency; Increased health needs.	‘*We're living on my son's expenses; I feel ashamed to add to his burdens. I feel anxious about this because he has a family’* (UP, female, 71–79 age group) ‘*Older people bear disease symptoms to save their dignity’* (SP, more than 10 years of experience) ‘*They hide their symptoms until the case gets really deteriorated, which incurs additional expenditures’* (FMU, female, 41–50 age group)
Humiliation and status regression	Obligation to attend in the absence of other alternatives; Staff's unprofessional attitude; Stigma; Favouritism;	‘*When someone is in need, and cannot pay, he's going to be more and more humiliated, he will be hurt and must bear silently, he knows his situation*’ (NUP, female, 60–70 age group). ‘*We should not have been obliged to come to the primary care centre, they are humiliating us after all these years’* (UP, male, 60–70 age group) ‘*They feel ashamed to use cheap or free services’* (SP, up to 10 years of experience)
Discomfort and unfairness	Extensive waiting time; Influx of refugees; Discrepancy in services provided for refugees compared to Lebanese citizens; Overcrowded waiting rooms.	‘*I am not seeking medical examination, I cannot sit there for 3 or 4 h, I pee on myself! The waiting issue constrains me from going, it's hard’* (UP, female, ≥ 80 age group) ‘*You see older people waiting in overcrowded and noisy rooms with pregnant women and children’* (FMU, female, 41–50 age group) ‘*They blame us for providing a wider package of services for Syrian refugees’* (SP, up to 10 years of experience)
Anxiety and uncertainty	Discontinued provision of certain chronic medications and unavailability of others; Narrow spectrum of services and inability to afford care in private settings.	‘*We are anxious about procuring our medications; if we do not find them at the centre, we are obliged to procure them from the pharmacy at a very high cost’* (UP, male, ≥ 80 age group). ‘*We know other people who abstain from taking medications that are missing, which could be dangerous’* (FMNU, male, 30–40 age group) ‘*We used to have a program to support older people with disabilities, it stopped because of a lack of funds; beneficiaries call frequently and ask to resume such services as they are unable to afford care’* (SP, up to 10 years of experience)
Dependency	Long travel time and distance to reach the PHCC; Expensive or unavailable transportation; Lack of information about PHCC services through age‐friendly channels.	‘*You know, Koura is lacking common transportation. Ordering a taxi incurs high cost, especially after the economic crisis’* (UP, male, 71–79 age group) ‘*Sometimes older patients do not show up to appointments because there is no one to drive and accompany them’* (SP, up to 10 years of experience) ‘*There is a lack of advertisement and information on available services, my daughter called and asked about attending physicians’* (UP, female, 71–79 age group)

Abbreviations: FMNU = family member of non‐users, FMU = family member of users, NUP = non‐user participant, UP = user participants.

#### 3.4.2 Negative Experiences of Family Members

Some family members reported a feeling of guilt and blame for neglect because of seeking care for their parents at PHCCs whose services are perceived as low‐quality and designed for disadvantaged groups.

People gossip, they think that we are neglecting our parents if we seek care from primary care centres.

FMNU, Female, 41–50 age group

They also feel responsible and challenged to understand their parents' medical conditions and accurately communicate them to all care providers within a fragmented system that lacks care coordination and referral systems. Similar to older people, they reported discomfort, particularly because of long waiting times and overcrowding. They also perceive status regression because of the obligation to seek care from PHCCs.

I would need 4 to 5 h to get to see the physician, and I am leaving my old‐aged mom alone at home.

FMU, female, 51–65 age group

#### 3.4.3 Negative Experiences of Service Providers

Many service providers reported a powerless feeling because of the increased demand and dwindling funds. They need to deal with people's complaints about service discontinuity, which is out of their control.

They come to the pharmacy, they yell, they nag about partially receiving their medications, but what can I do for them? It's out of my control.

SP, up to 10 years of experience

They also experienced difficulty with assessing and explaining treatment regimens for older people who present sensory and cognitive limitations, especially when they come unaccompanied. Some physicians felt aggrieved because of the unfair remuneration at PHCCs, obliging them to commit only for a few hours per week. This results in overcrowding during their shifts, affecting their ability to provide complete care for older people.

How can the physician preserve the older person's medical rights if his own financial rights are not preserved?

SP, more than 10 years of experience

When I need to examine 30 or 40 patients per hour, I cannot even look at their eyes.

SP, more than 10 years of experience

## 4 Discussion

This study identifies factors and perceptions shaping older people's decisions to use PHCCs and examines their experiences accessing PHCCs by considering their own perspectives as well as those of family members and service providers. It also acknowledges the experiences of family members accessing PHCCs and those of service providers delivering PHC to older people within PHCCs.

The insights gained from this study revealed shared perceptions among participants. These include the perceived consequences of the economic crisis that increased the financial dependency, limited the access to private care, increased reliance on PHCCs and aggravated older people's feelings of being a burden. The lack of government support for older people, anxiety because of service discontinuity, squeezed clinical examinations and discomfort due to poor service organisation were common reported factors contributing to negative experiences. However, the findings also showed divergent views. For instance, service providers valued the support they provide for older people, who in return expressed that services at PHCCs are not meeting their expectations and reported feelings of humiliation. Users of PHCCs have better perceptions regarding the quality of services provided by PHCCs compared with non‐users. While some users showed gratitude for the support they received, others reported challenges undermining their dignity.

### 4.1 Decisions Related to Using Services Delivered by PHCCs

This study identifies different factors underlying the use or non‐use of services delivered through PHCCs and highlights a shift in reliance on these services following the economic crisis. The findings suggest that PHCCs constitute a preferred choice due to their affordable services and proximity, particularly for older people with functional limitations or limited family support. Additional factors encouraging the use of PHCC services include positive attitudes and behaviours of service providers, as well as familiar and simple service processes.

Socio‐economic status and unequal health coverage emerged as significant factors delineating the choice of care settings, with a predominant preference for private care as long as it remains affordable. Consistent with literature from LMICs [8, 34], this preference stems from a common perception that private healthcare offers better quality than PHCC services. Family members, having an essential role in healthcare‐seeking decisions [34], do not recommend PHCCs for their older parents. However, this pattern started changing since the onset of the economic crisis (2019), which restricted families' ability to afford private care and increased older people's vulnerabilities. This explains a nearly 100% surge in services delivered at PHCCs between 2019 and 2023 [22]. The obligation to use services perceived as lower quality due to economic constraints illustrates how public policies and socio‐economic factors perpetuate inequities in healthcare access [35].

The study also reveals an information gap constraining the use of PHCCs. In general, Lebanese people have negative perceptions about dispensaries [36]. Mixing between dispensaries and PHCCs might have a negative influence on the image and use of PHCCs. People in the aftermath of the economic crisis significantly need affordable preventive and therapeutic services offered at PHCCs. The lack of information about such services may lead to unmet needs and poor health outcomes, deepened health disparities, and poor healthcare system performance [37].

The main service sought at PHCCs was chronic medication provision, because of market shortages during the crisis [38] and price increase after the lift of governmental subsidy [39]. This high demand could also be linked to the characteristics of participants, confirming the high prevalence of chronic diseases among older people who present low to medium socio‐economic status. The unexpected surge in demand has complicated the supply of services, particularly medications, which poses particular risks for people who have long depended on PHCCs and have no alternatives. Such challenges risk aggravating healthcare inequities and exacerbating adverse health outcomes, lack of adherence to treatment regimens, and mental burden [40].

The rising transportation costs have further compelled older people to seek care at nearby PHCCs. The increased reliance on PHCCs adds further strains to an already under‐resourced PHC system struggling with diminished funds, partly because of ongoing global conflicts [12].

### 4.2 Experiences With Access to PHCCs

Exploring access experiences was important, especially since perceptions of healthcare encounters impact the sustainment of service use [41]. Findings highlight varying experiences across PHCCs. Despite being all affiliated with the MOPH, a discrepancy exists across centres regarding service delivery, organisation and funding [21], with implications on care experiences. Findings are aligned with evidence from LMICs suggesting that care experiences vary according to socio‐economic contexts and service delivery models [8].

For some participants, access to PHCCs constitutes a gateway to restored dignity and well‐being, which confirms the PHC value, especially during economic downturns [7]. While family members feel relieved to get affordable care, older people report enhanced autonomy and familiarity with processes, gratitude for receiving affordable and quality services, and a sense of being respected, heard and cared for. However, access to free or affordable care implies a burden, including discomfort and a perception of status regression reported by older people and family members who feel obliged to seek perceived low‐quality services. Family members feel guilty for seeking care from PHCCs, especially since some older people experience humiliation and unfairness. While negative experiences of older people with access to PHC in LMICs are common [8, 9], insights stemming from this study are grounded within the Lebanese context. This offers an opportunity for a people‐centred transformation of services to address healthcare inequities [42].

Experiences of older people and family members are interrelated, since family members either seek care for older people or interfere in healthcare‐seeking decisions [34]. Understanding the experiences of family members was essential since negative perceptions may either suppress access to PHCCs or deepen the humiliating feeling of being obliged to use low‐quality services. Older people and service providers' experiences also seem interconnected. Service providers experiencing unfair work conditions feel drained and powerless due to the increased demand complicated by short funds [12]. This may aggravate the existing risk of burnout and turnover [43], impact the quality of care [43], and affect the relational aspect, and thus, older people's experiences [2]. Interconnected experiences suggest that improving older people's access to PHCCs requires a comprehensive approach that simultaneously addresses the unique needs of older people, family members and service providers.

**Figure 2:**
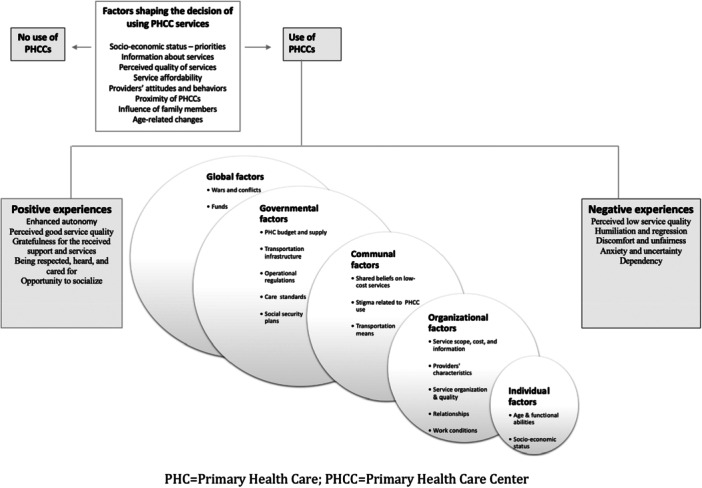
Older people's experiences with access to PHCCs.

Findings from this study converge with the work of Wolf et al. [2] to confirm that human experiences with healthcare are shaped by factors that surpass the individual and organisational levels. They also align with the socio‐ecological perspective, suggesting that health behaviours and experiences are influenced by factors at multiple levels [44]. We draw on this perspective to illustrate the complexity of factors shaping care decisions and experiences, as identified by participants (Figure 2).

At the individual level, experiences varied with the functional abilities, socio‐economic status and place of residence, highlighting the importance of social determinants of health in shaping healthcare access [45]. Older people presenting functional problems or a social role to fulfil reported negative experiences regarding the long waiting time, a factor perceived by other retired people as an opportunity to socialise. Individuals who felt obliged to use PHCC services because of their recent financial disadvantage reported more humiliating experiences compared to others who started seeking such services before the crisis. People living near PHCCs reported fewer challenges in reaching PHCCs compared to those who are distant. Similarly, the influence of geographical barriers [46] and socio‐economic disparities [35] on access to PHC in LMICs is well documented. Financial dependency induces a feeling of being a burden among older people, translated into delayed care and neglected preventive services, which are essential to manage comorbidities that prevail at older age [47].

At the organisational level, short and unstable funds remain key barriers to quality improvement, affecting service availability, affordability, approachability, organisation and quality [8]. As previously evidenced [3, 4], the relational aspect was of paramount importance. The unfair payment and overcrowding experienced by physicians are jeopardising the quality of care and threatening the sustainability of their work at PHCCs. This could aggravate existing staffing issues [21] and exhaust the remaining personnel, with critical implications on care quality and relationships [16]. In line with national data, administrators are challenged to manage the care delivery and operational costs with limited funds, which threatens the sustainment of their institutions [12]. Findings support evidence suggesting that enhancing the quality of care and users' experiences goes through caring for the workforce and ensuring fair pay, training opportunities and work prospects [2, 43].

At the community level, findings meet the descriptions by Wolf et al. [2] on the influence of social beliefs and shared experiences on older people's perception of care. PHCCs' services are perceived as below standard, and PHCC users are labelled as disadvantaged. This is exacerbating the feeling of humiliation among people who feel obliged to use such services following the economic crisis. Lacking transportation infrastructure and rising related costs add to older people's challenges with accessing PHCCs [46].

The governmental and global factors influence all other levels. For example, the lack of social pension plans [11] affects the socio‐economic status of individuals; the minimal investment in primary care and lack of financial support to PHCCs [21] exacerbate resource shortages and affect organisational processes and priorities; the exclusive reliance on NGOs to ensure chronic medications leads to care discontinuity. The reliance on external funds has contributed to the resilience of the PHC system during all crisis times [48]. However, the dependency on uncertain funds threatens the viability of PHC services [48], especially with global conflicts and wars imposing new emergencies leading to a shift of funds [12].

### 4.3 Implication for Policy and Practice

Understanding older people's experiences with access to PHCCs is key to improving the quality of care. Findings inform service providers and policymakers on aspects that should be reinforced across the individual, organisational, communal and governmental levels to generate more positive experiences. They call for inter‐sectoral collaboration to ensure the following: urgent pension plans; corrective actions to return bank deposits for older people; policies to ensure continuous provision of chronic medications at the primary care level; expanding the scope of PHC services to include home care and telehealth; standardising services across PHCCs; improving work conditions for the workforce and empowering them through training on geriatric care; reorganising care delivery models to reduce waiting time and overcrowd; and facilitating transportation of older people to PHCCs.

### 4.4 Strengths and Limitations

This study presents major strengths, including the involvement of different stakeholders, the adoption of various data collection methods, and its relevance and timely conduct, considering the increase in the older population and increased reliance on PHCCs' services. The study also presents limitations, including the limited volume of translated data, which restricts the full involvement of co‐authors in all data analysis steps. This was due to financial resources and data protection restrictions inherent in the ethical approval granted to the study. Recruitment methods excluded people with cognitive limitations and limited phone accessibility. This was mitigated through the involvement of family members who represented their views. The FGD method may have restricted individual responses, as some older people, in particular, may not be familiar with this method. Data inaccuracy might have been induced due to recall bias among participants, the social desirability response bias, and the possible mix of experiences across PHCCs and dispensaries. Data triangulation was useful to mitigate such biases. Due to the research design nature, findings are not generalisable but could be transferable to people sharing the same participants' characteristics.

## 5 Conclusion

Understanding the experiences of older people accessing PHCCs is necessary to transform practices and health systems. This study shows that access to PHCC is a choice for some older people and families and a gateway towards care and well‐being amidst the dire economic crisis. However, it remains an obligation for many others who lack alternatives and report negative experiences. Findings call for action at the organisational, communal, governmental and global levels to support Lebanese older people in accessing PHC and living with dignity. Quality improvement requires a multidimensional approach considering the needs of older people, family members and service providers. Researchers are invited to provide practitioners and policymakers with evidence‐based solutions that are rooted in different partners' perspectives through participatory approaches.

## Author Contributions

Saydeh Dableh, Thilo Kroll and Kate Frazer contributed to the conception of the study. All co‐authors contributed to the study design and methodology development. Saydeh Dableh provided resources, collected data and then analysed data with support from Thilo Kroll, Kate Frazer and Mathilde Azar. Thilo Kroll and Kate Frazer supervised the project. Saydeh Dableh wrote the first manuscript draft, which was revised and edited by all co‐authors who validated the results and approved the final manuscript version.

## Ethics Statement

This study was approved by the institutional research board of the University of Balamand in Lebanon (IRB‐REC/O/023‐23l0923, July 2023) and the health research ethics board of the University College Dublin (LS‐CO‐23‐185‐Dableh‐Kroll, 23 November 2023).

## Consent

All participants provided a signed informed consent and were informed about data confidentiality and security measures.

## Conflicts of Interest

We disclose that Dr. Kate Frazer [co‐author] is a member of the Health Expectations Editorial Board. There are no other disclosures to report.

## Supporting information

Supporting material 1‐ Table of themes, categories, codes and quotes.

Supporting material 2‐ The codebook.

## Data Availability

The data underlying this article are available in the article and its online supporting material. However, due to the nature of research and in compliance with GDPR requirements for data privacy, raw data, including audio recordings and participant identifiers, are not available.

## References

[1] E. Larson , J. Sharma , M. A. Bohren , and Ö. Tunçalp , “When the Patient Is the Expert: Measuring Patient Experience and Satisfaction With Care,” Bulletin of the World Health Organization 97, no. 8 (2019): 563–569, 10.2471/BLT.18.225201. 31384074 PMC6653815

[2] J. A. Wolf , V. Niederhauser , D. Marshburn , and S. L. LaVela , “Reexamining ‘Defining Patient Experience’: The Human Experience in Healthcare,” Patient Experience Journal 8, no. 1 (2021): 16–29, 10.35680/2372-0247.1594.

[3] O. Toritsemogba Tosanbami Omaghomi , A. Opeoluwa Akomolafe , O. Chinyere Onwumere , O. Ifeoma Pamela Odilibe , and E. Oluwafunmi Adijat Elufioye , “Patient Experience and Satisfaction in Healthcare: A Focus on Managerial Approaches—A Review,” International Medical Science Research Journal 4 (2024): 194–209, 10.51594/imsrj.v4i2.812.

[4] K. Browne , D. Roseman , D. Shaller , and S. Edgman‐Levitan , “Analysis & Commentary. Measuring Patient Experience as a Strategy for Improving Primary Care,” Health Affairs 29, no. 5 (2010): 921–925, 10.1377/hlthaff.2010.0238. 20439881

[5] A. Bitton , J. Fifield , H. Ratcliffe , et al., “Primary Healthcare System Performance in Low‐Income and Middle‐Income Countries: A Scoping Review of the Evidence From 2010 to 2017,” supplement, BMJ Global Health 4, no. S8 (2019): e001551, 10.1136/bmjgh-2019-001551. PMC6703296 31478028

[6] World Health Organization . Primary Health Care, accessed April 25, 2024, https://www.who.int/news-room/fact-sheets/detail/primary-health-care.

[7] World Health Organization . Building the Economic Case for Primary Health Care: A Scoping Review, accessed August 20, 2021, https://www.who.int/publications/i/item/WHO-HIS-SDS-2018.48.

[8] S. Dableh , K. Frazer , D. Stokes , and T. Kroll , “Access of Older People to Primary Health Care in Low and Middle‐Income Countries: A Systematic Scoping Review,” PLoS One 19, no. 4 (2024): e0298973, 10.1371/journal.pone.0298973. 38640096 PMC11029620

[9] G. Kelly , L. Mrengqwa , and L. Geffen , “‘They Don't Care About Us’: Older People's Experiences of Primary Healthcare in Cape Town, South Africa,” BMC Geriatrics 19, no. 1 (2019): 98, 10.1186/s12877-019-1116-0. 30947709 PMC6449977

[10] T. Motsohi , M. Namane , A. C. Anele , M. Abbas , and S. Z. Kalula , “Older Persons' Experience With Health Care at Two Primary Level Clinics in Cape Town, South Africa: A Qualitative Assessment,” BJGP Open 4, no. 3 (2020): bjgpopen20X101048, 10.3399/bjgpopen20X101048. PMC7465590 32605915

[11] Lebanese ministry of social affairs (MOSA) . The National Strategy for Older Persons in Lebanon 2020‐2030, accessed September 20, 2024, https://www.unescwa.org/sites/default/files/news/docs/online_-_final_english_strategy_for_online_use_1.pdf.

[12] R. S. Hamadeh , O. Kdouh , R. Hammoud , E. Leresche , and J. Leaning , “Working Short and Working Long: Can Primary Healthcare Be Protected as a Public Good in Lebanon Today?,” Conflict and Health 15, no. 1 (2021): 23, 10.1186/s13031-021-00359-4. 33827637 PMC8024932

[13] N. Kawa , J. Abisaab , F. Abiad , et al., “The Toll of Cascading Crises on Lebanon's Health Workforce,” Lancet Global Health 10, no. 2 (2022): e177–e178, 10.1016/S2214-109X(21)00493-9. 34800375

[14] E. Bou Sanayeh and C. El Chamieh , “The Fragile Healthcare System in Lebanon: Sounding the Alarm About Its Possible Collapse,” Health Economics Review 13, no. 1 (2023): 21, 10.1186/s13561-023-00435-w. 37014485 PMC10071460

[15] A. Shallal , C. Lahoud , M. Zervos , and M. Matar , “Lebanon Is Losing Its Front Line,” Journal of Global Health 11 (2021): 03052, 10.7189/jogh.11.03052. 33828836 PMC8005300

[16] F. El‐Jardali , R. Masri , and Z. Sleem . Lebanon's Economic Crisis by Sector: Reforming the Healthcare System. The Lebanese Center for Policy Studies, accessed September 18, 2024, https://www.lcps-lebanon.org/en/articles/details/4795/lebanon%E2%80%99s-economic-crisis-by-sector-reforming-the-healthcare-system.

[17] Z. Chemali , L. M. Chahine , and A. M. Sibai , “Older Adult Care in Lebanon: Towards Stronger and Sustainable Reforms,” Eastern Mediterranean Health Journal = La Revue de Sante de la Mediterranee Orientale = al‐Majallah al‐Sihhiyah li‐Sharq al‐Mutawassit 14, no. 6 (2008): 1466–1476, https://pubmed.ncbi.nlm.nih.gov/19161123/. 19161123

[18] A. M. Sibai , A. Rizk , and N. M. Kronfol , “Aging in Lebanon: Perils and Prospects,” Lebanese Medical Journal 63, no. 1 (2015): 2–7, 10.12816/0009912. 25906507

[19] S. Doocy , E. Lyles , B. Hanquart , and M. Woodman , “Prevalence, Care‐Seeking, and Health Service Utilization for Non‐Communicable Diseases Among Syrian Refugees and Host Communities in Lebanon,” Conflict and Health 10, no. 1 (2016): 21, 10.1186/s13031-016-0088-3. 27777613 PMC5070168

[20] N. M. Kronfol , “Access and Barriers to Health Care Delivery in Arab Countries: A Review,” Eastern Mediterranean Health Journal 18 (2012): 1239–1246, 10.26719/2012.18.12.1239. 23301399

[21] R. Hemadeh , O. Kdouh , R. Hammoud , T. Jaber , and L. A. Khalek , “The Primary Healthcare Network in Lebanon: A National Facility Assessment,” Eastern Mediterranean Health Journal 26, no. 6 (2020): 700–707, 10.26719/emhj.20.003. 32621505

[22] Lebanese Ministry of Public Health (MOPH) . Primary Health Care Annual Report, accessed January 30, 2025, https://www.moph.gov.lb/userfiles/files/HealthCareSystem/PHC/%D8%A7%D9%84%D8%AA%D9%82%D8%B1%D9%8A%D8%B1%20%D8%A7%D9%84%D8%B3%D9%86%D9%88%D9%8A%20%D9%84%D8%B4%D8%A8%D9%83%D8%A9%20%D8%A7%D9%84%D8%B1%D8%B9%D8%A7%D9%8A%D8%A9%20%D8%A7%D9%84%D8%B5%D8%AD%D9%8A%D8%A9%20%D8%A7%D9%84%D8%A3%D9%88%D9%84%D9%8A%D8%A9%20%D9%84%D9%84%D8%B9%D8%A7%D9%85%202023.pdf.

[23] Lebanese Republic Central Administration of Statistics . Labour Force and Household Living Conditions Survey 2018‐2019 in Koura, accessed October 24, 2023, http://www.cas.gov.lb/images/Publications/Labour_Force_District_Statistics/KOURA%20FINAL.PDF.

[24] J. W. Creswell , Qualitative Inquiry and Research Design: Choosing Among Five Approaches, 3rd ed. (SAGE, 2013).

[25] L. Doyle , C. McCabe , B. Keogh , A. Brady , and M. McCann , “An Overview of the Qualitative Descriptive Design Within Nursing Research,” Journal of Research in Nursing 25 (2020): 443–455, 10.1177/1744987119880234. 34394658 PMC7932381

[26] H. Kim , J. S. Sefcik , and C. Bradway , “Characteristics of Qualitative Descriptive Studies: A Systematic Review,” Research in Nursing & Health 40, no. 1 (2017): 23–42, 10.1002/nur.21768. 27686751 PMC5225027

[27] M. Sandelowski , “Whatever Happened to Qualitative Description?,” Research in Nursing & Health 23, no. 4 (2000): 334–340, 10.1002/1098-240x(200008)23:4<334::aid-nur9>3.0.co;2-g. 10940958

[28] B. C. O'Brien , I. B. Harris , T. J. Beckman , D. A. Reed , and D. A. Cook , “Standards for Reporting Qualitative Research: A Synthesis of Recommendations,” Academic Medicine 89, no. 9 (2014): 1245–1251, 10.1097/ACM.0000000000000388. 24979285

[29] K. Malterud , V. D. Siersma , and A. D. Guassora , “Sample Size in Qualitative Interview Studies: Guided by Information Power,” Qualitative Health Research 26, no. 13 (2016): 1753–1760, 10.1177/1049732315617444. 26613970

[30] M. M. Hennink , B. N. Kaiser , and V. C. Marconi , “Code Saturation Versus Meaning Saturation: How Many Interviews Are Enough?,” Qualitative Health Research 27, no. 4 (2017): 591–608, 10.1177/1049732316665344. 27670770 PMC9359070

[31] D. Abfalter , J. Mueller‐Seeger , and M. Raich , “Translation Decisions in Qualitative Research: A Systematic Framework,” International Journal of Social Research Methodology 24, no. 4 (2020): 469–486, 10.1080/13645579.2020.1805549.

[32] C. Erlingsson and P. Brysiewicz , “A Hands‐On Guide to Doing Content Analysis,” African Journal of Emergency Medicine 7, no. 3 (2017): 93–99, 10.1016/j.afjem.2017.08.001. 30456117 PMC6234169

[33] L. S. Nowell , J. M. Norris , D. E. White , and N. J. Moules , “Thematic Analysis: Striving to Meet the Trustworthiness Criteria,” International Journal of Qualitative Methods 16 (2017): 1–13, 10.1177/1609406917733847.

[34] R. J. Lilford , B. Daniels , B. McPake , et al., “Supply‐Side and Demand‐Side Factors Affecting Allopathic Primary Care Service Delivery in Low‐Income and Middle‐Income Country Cities,” Lancet Global Health 13 (2025): e942–e953, https://www.thelancet.com/pdfs/journals/langlo/PIIS2214-109X(24)00535-7.pdf. 40288402 10.1016/S2214-109X(24)00535-7

[35] Q. Gao , et al., “Inequalities in Older Age and Primary Health Care Utilization in Low‐ and Middle‐Income Countries: A Systematic Review,” International Journal of Health Services 52, no. 1 (2022b): 99–114, 10.1177/00207314211041234. 34672829 PMC8645300

[36] F. El‐Jardali , R. Fadlallah , and L. Matar , PRIMARY HEALTH CARE SYSTEMS (PRIMASYS) Comprehensive Case Study From Lebanon (WHO Alliance for Health Policy and Systems Research, 2017), https://iris.who.int/server/api/core/bitstreams/1bbc954c-48af-4c97-b19b-824391c84bb3/content.

[37] L. Shi , “The Impact of Primary Care: A Focused Review,” Scientifica 2012 (2012): 432892, 10.6064/2012/432892. 24278694 PMC3820521

[38] M. Cherfane , M. Boueri , E. Issa , et al., “Unveiling the Unseen Toll: Exploring the Impact of the Lebanese Economic Crisis on the Health‐Seeking Behaviors in a Sample of Patients With Diabetes and Hypertension,” BMC Public Health 24, no. 1 (2024): 628, 10.1186/s12889-024-18116-6. 38413883 PMC10900622

[39] M. Fleifel and K. Abi Farraj , “The Lebanese Healthcare Crisis: An Infinite Calamity,” Cureus 14, no. 5 (2022): e25367, 10.7759/cureus.25367. 35769680 PMC9235031

[40] J. M. Phuong , J. Penm , B. Chaar , L. D. Oldfield , and R. Moles , “The Impacts of Medication Shortages on Patient Outcomes: A Scoping Review,” PLoS One 14, no. 5 (2019): e0215837, 10.1371/journal.pone.0215837. 31050671 PMC6499468

[41] J. A. Ford , G. Wong , A. P. Jones , and N. Steel , “Access to Primary Care for Socioeconomically Disadvantaged Older People in Rural Areas: A Realist Review,” BMJ Open 6, no. 5 (2016): e010652, 10.1136/bmjopen-2015-010652. PMC4874140 27188809

[42] N. Mirza , E. Brown , and W. Hulko , “Service Users' Views on the Restructuring of Primary and Community Care Services for Older Adults: A Scoping Review,” Journal of Rural & Community Development 17, no. 3 (2022): 83–104, https://journals.brandonu.ca/jrcd/article/view/2037/585.

[43] M. Alameddine , M. Baroud , S. Kharroubi , et al., “Investigating the Job Satisfaction of Healthcare Providers at Primary Healthcare Centres in Lebanon: A National Cross‐Sectional Study,” Health & Social Care in the Community 25, no. 6 (2017): 1805–1816, 10.1111/hsc.12454. 28627051

[44] W. Kennedy , R. Fruin , A. Lue , and S. W. Logan , “Using Ecological Models of Health Behavior to Promote Health Care Access and Physical Activity Engagement for Persons With Disabilities,” Journal of Patient Experience 8 (2021): 23743735211034031, 10.1177/23743735211034031. 34350340 PMC8295941

[45] K. Vrtikapa , F. Hoque Urmy , and F. Hoque , “Social Determinants of Health: The Impact of This Overlooked Vital Sign,” Journal of Brown Hospital Medicine 4, no. 3 (2025): 138072, 10.56305/001c.138072. 40612083 PMC12224330

[46] D. Kwaitana , F. Chisoni , D. van Breevoort , et al., “Primary Healthcare Service Delivery for Older People With Progressive Multimorbidity in Low‐ and Middle‐Income Countries: A Systematic Review,” Transactions of the Royal Society of Tropical Medicine and Hygiene 118, no. 3 (2024): 137–147, 10.1093/trstmh/trad068. 37795606

[47] H. Wang , M. Naghavi , C. Allen , et al., “Global, Regional, and National Life Expectancy, All‐Cause Mortality, and Cause‐Specific Mortality for 249 Causes of Death, 1980‐2015: A Systematic Analysis for the Global Burden of Disease Study 2015,” Lancet 388, no. 10053 (2016): 1459–1544, 10.1016/s0140-6736(16)31012-1. 27733281 PMC5388903

[48] N. Aoun and M. Tajvar , “Healthcare Delivery in Lebanon: A Critical Scoping Review of Strengths, Weaknesses, Opportunities, and Threats,” BMC Health Services Research 24, no. 1 (2024): 1122, 10.1186/s12913-024-11593-w. 39334362 PMC11429949

