# Supplementary material for: The Experience of Accessing Primary Healthcare Centres in a Lebanese Community: Perspectives of Older People, Family Members and Service Providers
Source: Health Expect. 2025 Sep 27;28(5):e70449. doi: 10.1111/hex.70449 (PMC12476029; doi:10.1111/hex.70449)
Supplement: Supplementary file 1 — Supporting material 1‐ Table of themes, categories, codes and quotes. [file HEX-28-e70449-s001.docx]

**Supplementary material 1: Table of themes, categories, and codes with supportive quotes**

| **Theme** | **Category** | **Codes/supportive quotes** |
| --- | --- | --- |
| Perceptions shaping older people’s decision to seek care from PHCCs | Personal factors | **Perceived misconceptions about the PHC network**  *“I never heard about primary health care”* (UP, female, 71-79 age group)  *“Dispensaries are more common, I am not sure if primary health care centres exist in Lebanon”* (FMNU, female, 51-60 age group)  *“Primary health care includes all services needed throughout the lifespan, all services that affect one’s health including hospitalization, medical consultation, medication, tests, simple surgeries”* (UP, female, 71-79 age group).  “*This is the first time we learn such information about primary care services*” (NUP, female, 71-79 age group)  *“The dispensary is a primary health care centre” (FMNU, female, 41-50 age group)*  *“We have no idea about the difference between a dispensary and a primary care centre” (NUP, male,* ≥80 age group*)*  *“Primary health care centres do not exist in Lebanon” (FMNU, female, 41-50 age group)*  *“Dispensaries are more common; I am not sure if primary health care centres exist in Lebanon” (FMNU, female, 51-65 age group)*  “*They provide same services, but primary health care concept surpasses religious and sectarian character of dispensaries which is important for beneficiaries*” (NUP, male, ≥80 age group)  “A *dispensary provides ambulatory services while a primary health care centre includes in-patient services and long stay”* (FMU, female, *41-50 age group*)  “*PHCCs are all connected through an IT system. Any PHCC can access data about users in other PHCCs. They are affiliated with MOPH who apply control on all processes including medication distribution and service fee threshold; dispensaries work independently and lack control”* (SP, up to 10 years of experience).  **Perceived changes in circumstances**  *“Before the crisis is unlike after it”* (NUP, female, 71-79 age group).  *“I use the primary care centre because my husband passed away and his pension is stuck at the bank, I cannot even afford a loaf of bread, I am ruined!”* (UP, female, ≥80 age group)  *“There were middle and below middle social classes, now they’re gone, employees became like all other poor people”* (FMU, female, 30-40 age group)  *“They even stole our savings, this is unacceptable, unacceptable!”* (UP, male, 71-79 age group)  *“For those who used to visit physicians at their private clinics and are shifting now to services provided by our centres, it won’t be the same for them, their medications will be different, the physician won’t be able to spoil them”* (SP, more than 10 years of experience)  *“Now amidst this financial hardship and high cost of medications, one is breaking his ego and coming to primary care centres”* (FMNU, female, *41-50 age group*)  *“They have shyness, they used to have a good economic status and now they are obliged to come to the centre”* (SP, more than 10 years of experience)  *“An army commander came, he has never been into a centre, now he used to come, school principals came, we didn’t use to see those people, and then, by little steps, it became normal, now they do not have a problem, they cannot afford care in other settings, what can they do?”* (SP, more than 10 years of experience)  *“I used to do a yearly check-up, to get reassured about my health, but now no, currently no, no I can’t afford them”* (UP, male, 71-79 age group).  **Perceived difference between PHCCs and private clinics**  *“When I go to the hospital outpatient clinic, they welcome me warmly. When I went to the medical centre, she made me feel indebted for the favour of providing services”* (FMNU, male, 30-40 age group)  *“At private clinics you go on time, get examined on time, the physician provides enough time to talk to him, joke with him, however at the medical centre, no the physician does not have much time for him”* (FMNU, female, *41-50 age group*)  *“At primary care centres they do what is strictly necessary; if you present high blood pressure, they measure it and prescribe medications. Questions about since when it happened and how it happened do not exist. There is no time, the physician is extremely overwhelmed because he attends the centre once a week and there is a high demand, you know, he does not have time, indeed!”* (FMU, female, *51-65 age group*)  *“You need to ask them about your body, but you can’t. They’re in rush*” (NUP, male, 71-79 age group)  *“The physician looks at the most important issues, the patient asks one question, that’s over, next”* (SP, up to 10 years of experience)  *“A considerable part of the treatment consists of talking, explaining the condition until the patient is convinced to adhere to treatment, and this takes time. I provide this at my clinic, but I cannot at primary care centres”* (SP, up to 10 years of experience)  “*When examining people with multiple sclerosis, they need the physician to explain. If I need to explain to them at the PHCC I need one hour, I won’t explain properly. At the clinic, yes, I do, and I answer all questions, they usually have a lot”.* (SP, more than 10 years of experience)  *“The whole visit at the PHCC is worth one examination at his clinic”* (NUP, female, 71-79 age group)  *“It is not possible that a patient comes to my private clinic, pay 30$, and see him for 5 minutes and let him go. I need to talk to him a lot, ask him, and then I examine him normally as I do anywhere”.* (SP, more than 10 years of experience)  *“How can the physician preserve the older person’s medical rights if his own financial rights are not preserved?”* (SP, up to 10 years of experience)  *“The doctor, not all of them, does not give the appropriate time for the patient, and deals differently with the patient at private clinics”* (SP, up to 10 years of experience)  **Shared decision-making**  *“I called my son to discuss with him my neighbour’s recommendation for benefiting of services delivered by the nearby centre, but he totally refused”* (NUP, Female, 71-70 age group*)*  “*My daughter does not want me to go to the centre; she fears gossiping*” (NUP, Female, 71-70 age group) |
|  | Contextual factors | **Perceived consequences of the economic crisis**  *“They say look at our previous situation and where we got nowadays, they cry”* (FMU, female, 30-40 age group)  *“Now we became nothing, zero!”* (UP8, female, 71-79 age group).  “*Mr. the army commander and myself have the same case, we used to have a 100% health coverage and many other benefits; we should not have been obliged to come to the primary care centre, they are humiliating us after all these years”* (UP, male, 60-70 age group)  “*Now we become afraid of getting hospitalized*” (UP8, female, 71-79 age group).  “*There is nothing that can help in covering healthcare service fees, this is a real problem”* (NUP, female, 71-79 age group).  “*If one’s is lacking external support, he cannot live and sustain, at all. This is my personal experience. This is an essential factor. If this is my case, what to say about those who have no one to provide support? What is their situations?!* (NUP, male, 71-79 age group).  *“I hope for our situation to change, for us to take a breath again and feel that there is someone who cares about us”* (UP8, female, 71-79 age group).  **Perceived lack of government support**  *“In Lebanon, older people are left to their unknown fate. The government should take care of us, especially that we have spent our lives serving the society and have given with hearts. We deserve at our old age to get decent services”* (NUP, male, 60-70 age group)  *“An older person has given a lot, got tired and did not get anything in return. His money is gone and we are trying at our centres, with primitive resources, to cover some of his needs”* (SP, more than 10 years of experience) |
| The burden of free care delivered at PHCCs | Negative older people’s experiences | **Perceived low quality**  *“It seems that the service quality is not zero, it’s below zero”* (UP, male, ≥80 age group)  “*The physician is seeing around 40 patients in one hour or one hour and a half, he deals with you as a number”* (NUP, female, 71-79 age group)  *“I didn’t go, but I hear from those who use their services that quality is low, and they do not find their expensive medications”* (NUP, male, 60-70 age group)  *“It’s a trust issue, even when the same physician goes to the centre, it is not the same, you feel that at his clinic, you have more time, you can ask more, you feel relaxed, and he is more meticulous”* (NUP, female, 60-70 age group).  “*They want you to leave so that someone else comes in. It happened to me, he is good, but it's all fast, fast. They are running out of time, and they need to handle many cases, at many places*” (UP, female, 71-79 age group).  *“At the centres, people are just numbers, numbers, numbers… It is more like passing numbers quickly, no matter how, without special attention*” (NUP, female, 71-79 age group)  “Quality *depends on the physician, trust, morals, values*” (NUP, male, 71-79 age group)  “At primary care centres, physicians do what is strictly necessary, there is no time for discussions” (FMU, male, 30-40 age group)  *“For sure I won’t be satisfied with a physician who do not discuss and explain. I don’t accept to be submissive; it is my right to know all details related to the physician’s decisions about my health but unfortunately, that does not happen often”* (NUP, male, ≥80 age group)  *“You see older people waiting in overcrowded and noisy rooms with pregnant women and children”* (FMU, female, 41-50 age group)  *“For certain specialties, they wait for long and they nag, the demand is high and sometimes the physician arrives late”* (SP, up to 10 years of experience)  *“Honestly, I have never come across a doctor who elaborates on these matters. He always focuses on your disease and prescribes treatment, but he doesn’t go into detail such if you have a family support, your life, how you feel, how you deal psychologically with events and your illness. It rarely happens. We lack this culture in Lebanon”* (UP, male, 71-79 age group)  “*He got mad [the physician] because I was asking about the treatment; he gave me the prescription paper and pen and told me: you prescribe!”* (UP, male, 71-79 age group)  *“They blame us for providing a wider package of services for Syrian refugees”* (SP, up to 10 years of experience)  **Felling of being a burden**  *“We’re living on my son’s expenses; I feel ashamed to add to his burdens, I feel anxious about this because he has a family”* (UP, female, 71-79 age group)  *“Older people feel like a burden because of their lack of financial resources but mostly because of their increasing health needs”* (NUP, male, 71-70 age group)  *“Older people skip preventive care, like cancer screening tests, because of other priorities and limited financial resources”* (SP, up to 10 years of experience)  *“They hide their symptoms until the case gets really deteriorated, which incur additional expenditure”* (FMU, female, 41-50 age group)  *“Older people in Lebanon say that they are a burden on the shoulder of their families”* (FMNU, male, 50-60 age group)  “*Older people bear as much as they can these days*” (UP, female71-79 age group).  *“Older people bear disease symptoms to save their dignity”* (NUP, male, 71-79 age group)  *“This age is very difficult”* (NUP, female, 71-79 age group)  *“It was a simple cough and cold, we begged him to go to the physician, he kept on saying it will pass, until he developed pneumonia. He needed cortisone, injections, and many other things. He didn’t want to bother us at the beginning but the hassle and expenses were greater at the end”* (FMNU, female, *41-50 age group*)  “*99% of Lebanese people seek care at a final stage”* (NUP, male, 60-70 age group)  **Humiliation and status regression**  *“When someone is in need, and cannot pay, he’s going to be more and more humiliated, he will be hurt and must bear silently, he knows his situation*” (NUP, female, 60-70 age group).  “I*f you go without calling, they question you and reproach you… the way they welcome you makes you feel humiliated, you won’t like to go there anymore*” (NUP, female, 60-70 age group).  *“We should not have been obliged to come to the primary care centre; they are humiliating us after all these years”* (UP, male, 60-70 age group).  *“You feel like a beggar here”* (NUP, female, 60-70 age group).  *“The Lebanese proverb says: beggars can’t be choosers”* (UP, male, 71-79 age group).  “*They will let you line up to get medications, you feel humiliated*” (NUP, female, 60-70 age group).  *“Regarding the waiting time, we are not satisfied but we are obliged”* (UP, female, 71-79 age group).  “*When services are not well-organized people won’t accept them, even when in need for them, he feels humiliated, he would say I preserve my dignity and go to private clinic, no matter what”* (NUP, male, 71-79 age group).  **Discomfort**  *“I am not seeking medical examination, I cannot sit there for 3 or 4 hours, I pee on myself! The waiting issue constrains me from going, it’s hard”* (UP, female, ≥80 age group)  “*Those days we became in need to go there to get medications, but because of the influx of strangers and overcrowding, we’re not being able to get it, the quantity is finished while we arrive, and this prevails in all medical centres*” (NUP, female, 71-79 age group).  *“Mothers with 2 or 3 children, running after them, the place is narrow, and the older person gets upset”* (UP, female, ≥80 age group).  “*I never go there when it is overcrowded, honestly, I can’t”* (UP8, female, 71-79 age group).  “*Lebanese are considered minority among foreigners in the centre”* (NUP, female, 71-79 age group).  “*When I come to the centre and I see this overcrowd and chaos, and non-Lebanese people are also seeking medications, it makes me feel that the centre is not appropriate for me, I won’t come back”* (UP, male, 71-79 age group).  **Anxiety and uncertainty**  *“We are anxious about procuring our medications; if we do not find them at the centre, we are obliged to procure them from the pharmacy at a very high cost”* (UP, male, ≥80 age group).  *“We know other people who abstain from taking medications that are missing, which could be dangerous”* (FMNU, male, 30-40 age group)  *“We used to have a program to support older people with disabilities, it stopped because of lacking fund; beneficiaries call frequently and ask to resume such services as they are unable to afford care”* (SP, up to 10 years of experience)  *“The older person lives with an obsessive idea, a constant mental distress: what do I do if any health problem happens to me?!”* (UP, male, 71-79 age group)  **Dependency**  *“You know, Koura is lacking common transportation. Ordering a taxi incurs high cost especially after the economic crisis”* (UP, male, 71-79 age group)  *“Sometimes older patients do not show up to appointments because there is no one to drive and accompany them”* (SP, up to 10 years of experience)  *“There is a lack of advertisement and information on available services, my daughter called and asked about attending physicians”* (UP, female, 71-79 age group) |
|  | Negative providers’ experiences | **Powerlessness**  *“No one can imagine the challenges we’ve been through during the past year, we had to deal with extreme cases, older people who have no one, nothing, and we managed with the minimal resources that we had”* (SP, up to 10 years of experience)  *“It’s difficult to make diagnosis for patients who do not benefit of diagnostic exams anymore and cannot pay for them at private settings”* (SP, more than 10 years of experience).  *“They come to the pharmacy, they yell, they nag about partially receiving their medications, but what can I do for them? It’s out of my control”* (SP, up to 10 years of experience)  **Difficulty to asses and explain treatment regimens**  *“How can the physician preserve the older person’s medical rights if his own financial rights are not preserved*?” (SP, more than 10 years of experience)  *“When I need to examine 30 or 40 patients per hour, I cannot even look at their eyes”* (SP, more than 10 years of experience). |
|  | Negative family members’ experiences | **Discomfort**  *“I would need 4 to 5 hours to get to see the physician and I am leaving my old-aged mom alone at home”* (FMU, female, 51-65 age group)  **Perceived regression**  “*Seeking care from a PHCC is perceived by the society members as a step-down”* (FMNU, male, 30-40 age group)  *“Society consider it [seeking care from a PHCC] as a disaster”* (FMNU, female, 41-50 age group)  **Guilty and blamed for negligence**  *“I feel guilty for not being able to provide the best quality care for my father”* (FMU, female, 41-50 age group)  *“People gossip, they think that we are neglecting our parents if we seek care from primary care centres*” (FMNU, Female. 41-50 age group)  **Responsible for coordinating care for their parents**  “*The family member needs to know the case to explain to the physician”* (FMNU, female, 41-50 age group)  “*You need to be a half-doctor to memorize all medical details and share them through different providers”* (FMNU, female, 41-50 age group) |
| Accessing PHCCs: A gateway to restored dignity and well-being | Positive older people’s experiences | **Enhanced autonomy**  *“I prefer going to the health centre, it is nearby and I can go alone”* (UP, female, 71-79 age group)  *“The staff member explained that I just need to call ahead of time to book an appointment”* (UP, male, 71-79 age group)  **Perceived good service quality**  *“The quality of services is as good as those delivered through private clinics”* (UP, female, 60-70 age group).  *“Physicians who attend at the centre are well-known and have their private clinics”* (UP, male, 60-70 age group).  *“As in private clinics, a nurse prepares you and accompanies you to the physician who dedicates appropriate time”* (FMU, female, 60-70 age group).  **Gratitude for the received support and services**  *“We are grateful for getting the medications that we need”* (UP, male, 60-70 age group).  “*A physician and a nurse come to my home and bring medications for free, otherwise, I would not be able to get care”* (UP, female, 60-70 age group).  **Feeling respected, heard, and cared for**  *“People at primary care centres are very good and friendly. They respect older people specifically”.* (UP, female, 60-70 age group).  *“Physicians provide adequate time, they listen”.* (UP, female, 60-70 age group)  “*Once they get beneficiaries using a wheelchair, they give them a pass, they do not wait a minute”* (FMU, female, 41-50 age group).  **Opportunity to socialize**  *“I go there every month, I am used to doing and I consider staff as my children”* (UP, female, 71-79 age group).  *“They ask me questions about my lifestyle and I share with them my habits and concerns; I appreciate staff when they actively listen to me and provide counselling”* (UP, female, 60-70 age group). |
|  | Positive providers’ experiences | **Pride to support older people during the crisis;**  *“We are proud for being able to help older people during the most difficult times”* (SP, up to 10 years of experience).  *“We did our best to support hopeless people; with the minimum resources we had, me were able to make a difference”* (SP, up to 10 years of experience).  **Rewarding to see older people’s gratitude**  *“We went through tough times during the past period but their gratitude for the provided help was rewarding”* (SP, up to 10 years of experience)  *“We forget all struggles when they get satisfied of the received support”* (SP, up to 10 years of experience)  *“We are really proud, despite all challenges and short resources, we managed and we helped older people who had no one, nothing! But their grateful attitude was rewarding”* (SP, up to 10 years of experience) |
|  | Positive family members’ experiences | **Relief to get affordable care**  *“It could be a disaster without services provided by the centre at low cost*” (FMU, male, 30-40 age group)  *“The primary care centres are offering services at affordable prices; we have no other options”* (FMU, female, 41-50 age group)  **Respectful relationships**  “*They used to call me every six months to remind me about my father’s checkup, they are really kind”* (FMU, female, 30-40 age group)  “*It really makes a difference when staff treats you respectfully”* (FMU, female, 30-40 age group) |
