# Supplementary material for: The Experience of Accessing Primary Healthcare Centres in a Lebanese Community: Perspectives of Older People, Family Members and Service Providers
Source: Health Expect. 2025 Sep 27;28(5):e70449. doi: 10.1111/hex.70449 (PMC12476029; doi:10.1111/hex.70449)
Supplement: Supplementary file 2 — Supporting material 2‐ The codebook. [file HEX-28-e70449-s002.docx]

| Supplementary Material 2: The codebook **Theme 1. Perceptions shaping older people’s decision to seek care from PHCCs** | |
| --- | --- |
| **Code** | **Description as per participants accounts** |
| **Category 1.1. Personal factors** | |
| Perceived misconceptions about the PHC network | Refers to participants manifesting unfamiliarity with the PHC concept and using PHCCs and dispensaries interchangeably without being able to identify the difference between the two types of settings. |
| Perceived changes in circumstances | Refers to the marital, financial, and physiological changes that happen with age but also to the changing attitude towards the use of PHCC services, after the economic crisis. |
| Perceived difference between PHCCs and private clinics | Refers to reported difference regarding the quality of services delivered by the private sector compared to services delivered by PHCCs. |
| Shared decision-making | Reflects the influence of relatives, notably children on older people’s decisions regarding the choice of the care setting. |
| **Category 1.2. Contextual factors** | |
| Perceived consequences of the economic crisis | Denotes the major consequences of the economic crisis on older people’s health needs and behaviours, on their financial status and their health coverage. |
| Perceived lack of government support | Refers to Lebanese OP comparing their life conditions to those of OP living abroad and blaming the government for being responsible of their bad situation. |

**Theme 2. Accessing PHCCs:** **A gateway to restored dignity and well-being**

| **Code** | **Description as per participants accounts** |
| --- | --- |
| **Category 2.1. Positive older people’s experiences** | |
| Enhanced autonomy | Describes the reported independency of older people to reach nearby PHCCs alone, and the easiness of making appointments. |
| Gratitude for the received support and services | Denotes the feeling reported by older people in relation to retrieving, at PHCCs, services that they need at affordable prices, including chronic medications, home care,  supportive paramedical equipment, and periodic tests. |
| Perceived good service quality | Refers to older people reporting good quality of services because of covering their needs, the providers’ competence, and the implementation of patient preparation as part of the medical examination. |
| Feeling respected, heard, and cared for | Denotes the satisfaction of older people with the welcoming and caring staff and providers’ behaviours, their positive communication, active listening, equal treatment of people with prioritisation of older people who present specific cases and needs. |
| Opportunity to socialize | Describes simple processes and familiarity of older people with staff members and other beneficiaries. |
| **Category 2.2. Positive providers’ experiences** | |
| Pride to support older people during the crisis; | Refers to the reported feeling of pride for deploying efforts to support older people during the crisis despite resource shortage. |
| Rewarding to see older people’s gratitude | Refers to experiencing satisfaction when older people show gratitude for the provided support and services |
| **Category 2.3. Positive family members’ experiences** | |
| Relief to get affordable care | Describes the sense of relief for getting affordable healthcare services and avoiding out of pocket health expenses. |
| Respectful relationships | Denotes the positive attitudes and behaviours of staff and healthcare professionals and their impact on fostering positive relationships with users. |

**Theme 3: The burden of free care delivered at PHCCs**

| **Code** | **Description as per participants accounts** |
| --- | --- |
| **Category 3.1. Negative older people’s experiences** | |
| Perceived low quality | Describes the reported perception of low service quality based on the correlation with low cost, the superficial medical examination, the providers’ competence, the physicians’ communication skills, and the shared negative experiences. |
| Perceived burdensome feeling among older people | Describes the burdensome feeling of older people resulting from their financial and physical dependability of other family members. |
| Humiliation and status regression | Denotes the feeling resulting from the staff unprofessional attitude, the obligation to seek PHCCs’ services because of the financial shortage among people who used to have health coverage, and stigmatization of PHCC users for being disadvantaged. |
| Discomfort and unfairness | Refers to the experiences resulting from the long waiting time at PHCCs to get services, the overcrowded and uncomfortable waiting rooms, the perceived difference in care delivery and service cost compared to refugees, |
| Anxiety and uncertainty | The feeling reported by older people regarding the discontinuous provision of their chronic medications at PHCCs and the possible need to use expensive services delivered at private settings because of the narrow spectrum of services delivered at PHCCs. |
| Dependency | Denotes the need of older people for transportation assistance to reach PHCCs because of long travel time and distance or the deficient common transportation system. |
| **Category 3.2. Negative providers’ experiences** | |
| Powerlessness | Refers to the experience of providers resulting from demand for services that exceeds the available resources. |
| Difficulty to asses and explain treatment regimens | Refers to reported cases when providers experience difficulty to get accurate information during assessment or explain treatment directions for older people with sensory and cognitive limitations who present alone to the PHCC. |
| **Category 3.3. Negative family members’ experiences** | |
| Discomfort | Denotes experiences reported by family members who wait in overcrowded waiting rooms and for long to get services for their parents. |
| Perceived regression | Describes the feeling reported by family members who became obliged to seek care from PHCCs because of financial shortages. |
| Guilty and blamed for negligence | Describes the feeling of family members for taking the decision to seek care for their parents from PHCCs. It denotes personal and social aspects. |
| Responsible for coordinating care for their parents. | Describes the experience of family members who need to understand their older relatives’ medical cases and details and transmit them across care providers because of the absence of appropriate care coordination and referral systems. |
